# The effect of Propofol versus Dexmedetomidine as anesthetic agents for oocyte pick-up on in vitro fertilization (IVF) outcomes

**DOI:** 10.1038/s41598-021-03177-z

**Published:** 2021-12-14

**Authors:** Özcan Budak, Mehmet Sühha Bostancı, AyçaTaş Tuna, Veysel Toprak, Hüseyin Çakiroğlu, Koray Gök

**Affiliations:** 1 Department of Histology and Embryology and Artificial Reproductive Techniques, Faculty of Medicine, Sakarya University, Sakarya, Turkey; 2 Department of Obstetrics and Gynecology and Artificial Reproductive Techniques, Faculty of Medicine, Sakarya University, Sakarya, Turkey; 3 Department of Anesthesiology and Reanimation, Faculty of Medicine, Sakarya University, Sakarya, Turkey; 4 Department of Obstetrics and Gynecology, Private Tatvan Can Hospital, Bitlis, Turkey; 5 Medical and Experimental Research Center, Faculty of Medicine, Sakarya University, Sakarya, Turkey; 6 Department of Obstetrics and Gynecology, Faculty of Medicine, Sakarya University, Sakarya, Turkey

**Keywords:** Medical research, Molecular medicine

## Abstract

This study aimed to evaluate the effects of propofol and dexmedetomidine over different timescales on the IVF outcomes for transvaginal oocyte retrieval (TVOR). Twenty-four rats included in the study were divided into two main groups and three subgroups were subjected to the ovulation induction process. Group 1 was administered propofol (100 mg/kg i.v.) and group 2 were administered dexmedetomidine (25 µg/kg i.p.) The oviduct collection procedure was completed within 15 min for subgroup Pro15min, Dex15min (n = 4), within 16 to 30 min for subgroup Pro30min, Dex30min (n = 4) and within 31 to 60 min for subgroup Pro60min, Dex60min (n = 4) after euthanasia. The total number of oocytes was counted. After in vitro fertilization, the number and quality of embryos were evaluated. The number of pups born were evaluated after embryo transfer. The embryo number, quality and pup count decreased as the administration time for propofol increased (p < 0.05). No statistically significant difference was found between the dexmedetomidine subgroups for embryo number, quality and pup count(p > 0.05). As the exposure time to propofol increased, the number and quality of embryos obtained, and the pup count, decreased. The use of dexmedetomidine had no negative impacts on the number of embryos, their quality or the number of pups.

## Introduction

In vitro fertilization (IVF) is currently the most widely used assisted reproductive technique worldwide. The IVF procedure technically includes ovarian stimulation and monitoring, oocyte collection with transvaginal follicle aspiration, fertilization in the laboratory and finally, transfer of embryos back to the uterus. Obtaining oocytes from ovarian follicles by ultrasound-guided needle aspiration from the vaginal wall requires anesthesia and/or analgesia, and this is defined as transvaginal oocyte retrieval (TVOR)^1^. Although TVOR is a relatively simple and minimally invasive procedure, the patient may experience anxiety and pain due to puncture of the vaginal skin mucosa and ovarian capsule with a needle. Various methods of anesthesia are used to manage the patients, such as general anesthesia, conscious sedation and regional anesthesia^2,3^. There is no consensus on the type of anesthetic agent to use for TVOR.

Several studies have indicated that anesthetic drugs can enter the follicular fluids (FF)^4^. There is some concern about anesthetic drugs accumulating in FF and their negative impacts on fertilization rates and embryo development under general anesthesia.

Propofol is a rapidly acting anesthetic agent with short induction and recovery times^5^. The most commonly used anesthetic agent during TVOR is intravenous propofol, which is a safe drug for use in IVF^1,6^.

However, some studies have revealed that propofol use is associated with reduced fertilization rates (FR) and inhibition of blastocyst development in single cell embryos^7^.

Dexmedetomidine is a centrally acting α-2 receptor agonist with sedative and analgesic properties without respiratory suppressing effects^8^. However, dexmedetomidine use may be limited by the increased incidence of hypotension and bradycardia, and a limited ability to achieve deep sedation^9^. There are not enough studies on the use of dexmedetomidine during the TVOR process.

This study aimed to evaluate the effect of these two anesthetic agents used in anesthesia for different durations on IVF results.

## Results

A total of 58 oocytes (50 metaphase II (MII), 4 metaphase I (MI) and 4 germinal vesicles (GV))—of which 44 fertilized—were collected in subgroup Pro15min. For subgroup Pro30min, 57 oocytes were obtained (49 MII, 5 MI, 3 GV), and 25 were fertilized. Fifty-six oocytes (49 MII, 3 MI, + GV) were obtained from subgroup Pro60min, and 11 of them were fertilized.

There were 31 s day embryos (SDE) in total (22 grade 1) from subgroup Pro15min, and 18 pups were obtained. For subgroup Pro30min, there were 14 s day embryos (SDE) in total (9 grade 1) and 6 pups were obtained. Eleven SDEs (2 grade 1) were from subgroup Pro60min, and 4 pups were obtained.

A total of 60 oocytes (51 MII, 5MI, 4GV) were collected in subgroup Dex15min, and 46 were fertilized. For subgroup Dex30min, 57 oocytes were obtained (51 MII, 2 MI, 4 GV) and were fertilized. Fifty-seven oocytes (47 MII, 6MI, 4 GV) were produced by subgroup Dex60min, and 11 of them were fertilized.

There were 34 SDEs (23 grade 1) from subgroup Dex15min, and 19 pups were obtained. For subgroup Dex30min, there was a total of 33 SDEs (25 grade 1) and 19 pups were obtained. Thirty-one SDEs (23 grade 1) were produced by subgroup Dex60min and 19 pups were obtained.

In our study, no statistically significant difference was found between the mean number of MII, MI, GV and total oocyte counts between the subgroups (P > 0.05) (Table 1).

**Table 1:** Comparison of oocyte maturation and fertilization numbers of propofol and dexmedetomidine groups.

Parameter	Promin15 (n = 4)	Promin30 (n = 4)	Promin60 (n = 4)	Dexmin15 (n = 4)	Dexmin30 (n = 4)	Dexmin60 (n = 4)	p value
M II	12.5 ± 0.58	12.25 ± 0.5	12.25 ± .96	12.75 ± 1.26	12.75 ± 0.5	11.75 ± 1.26	0.615
M I-MII	1 ± 0.82	1.25 ± 0.5	0.75 ± 0.5	1.25 ± 0.5	0.5 ± 0.58	1.5 ± 0.58	0.227
GV	1 ± 0.82	0.75 ± 0.5	1 ± 0.82	1 ± 0 .82	1 ± 0 .82	1 ± 0.82	0.995
Total oocyte count	14.5 ± 0.58	14.25 ± 0.5	14 ± 0	15.25 ± 0.96	14.25 ± 1.26	14.25 ± 0.5	0.301
Fertilized GV	0.50 ± 0.58	0 ± 0	00 ± 0	0.25 ± 0.5	0.5 ± 0.58	0.5 ± 0.58	0.347
Fertilized MI-II	1.750 ± 0.5	0.750 ± 0.5	0 ± 0	1.5 ± 0.5	1.25 ± 0 .5	1 ± 0.82	0.019
Fertilized MII	8.75 ± 0.5	5.5 ± 0.57	2.75 ± 0.5	9.75 ± 0.5	9.25 ± 0.58	9.25 ± 0.5	0.002*
Total number of fertilized oocytes	11 ± 0.82	6.25 ± 0.5	2.75 ± 0.5	11.5 ± 1	11.25 ± 0..96	11.5 ± 0.58	0.004*

*MII* metaphase II stage oocyte, *MI-II* matured from MI to MII oocyte, *GV* germinal vesicle, *Promin15* Propofol subgroup (0–15 min), *Promin30* Propofol subgroup (15–30 min), *Promin60* Propofol subgroup (30–60 min), *Dexmin15* Dexmedetomidine (0–15 min), *Dexmin30* Dexmedetomidine (15–30 min), *Dexmin60* Dexmedetomidine (30–60 min).

Groups were compared using the Kruskal Wallis test; paired comparisons were made using the Mann–Whitney U test in the parameters that differed (*P < 0.05).

**Figure 1:**
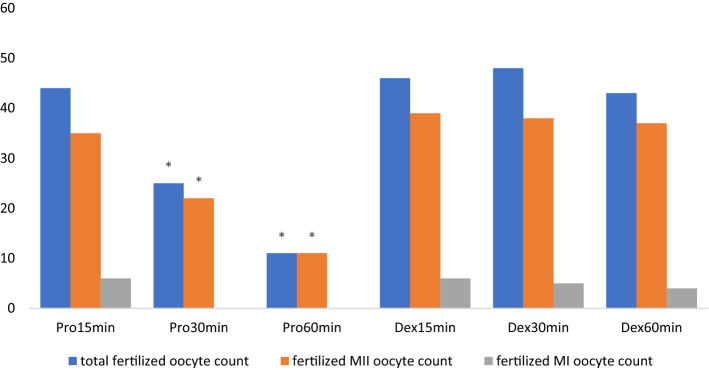
Comparison of fertilized MII oocyte and total fertilized oocyte counts between groups. *Pro15min* Propofol subgroup (0–15 min), *Pro30min* Propofol subgroup (15–30 min), *Pro60min* Propofol subgroup (30–60 min), *Dex15min* Dexmedetomidine (0–15 min), *Dex30min* Dexmedetomidine (15–30 min), *Dex60min* Dexmedetomidine (30–60 min). There was no fertilization in MI oocytes in groups Pro30min and Pro60min. The groups were compared using the Kruskal Wallis test; paired comparisons were made using the Mann–Whitney U test for the different parameters. (**P < 0.05).

Fertilized MII, MI–MII oocyte counts and total fertilized oocyte counts for the subgroups are presented in Fig. 1.

When the propofol groups were compared, there was a significant difference in terms of fertilized MII oocytes between the Pro15min subgroup, and the Pro30min and Pro60min subgroups (P = 0.017 and P = 0.015, respectively). This significant difference was also found in terms of the total fertilized oocyte count between the Pro15min subgroup, and the Pro30min and Pro60min subgroups (P = 0.017 for both).When the Pro30min and Pro60min subgroups were compared for the number of fertilized MII, MI and total fertilized oocytes, it was found to be significantly higher in the Pro30min group (P = 0.017, P = 0.040 and P = 0.015, respectively).

There was no statistically significant difference between dexmedetomidine subgroups in terms of fertilized MII, MI oocyte counts and total fertilized oocyte counts (P > 0.05) (Fig. 1).When the same time periods were compared, there was no statistically significant difference between the total fertilized oocyte count and fertilized MI oocytes between the Pro15min and Dex15min subgroups (P = 0.350 and P = 0.495, respectively); however, although the mean numbers of fertilized MII oocytes were close to each other, a statistically significant difference was found in favor of the Dex15min group (P = 0.040).When the Pro30min and Dex30min subgroups were compared in terms of fertilized MII, MI and total fertilized oocyte counts, statistically significant differences were found in favor of the Dex30min subgroup (P = 0.017 and P = 0.018, respectively).When the Pro60min and Dex60min subgroups were compared for the numbers of fertilized MII, MI and total fertilized oocytes, they were found to be statistically higher in the Dex60min subgroup (P = 0.015, P = 0.046 and P = 0.017, respectively).

**Figure 2:**
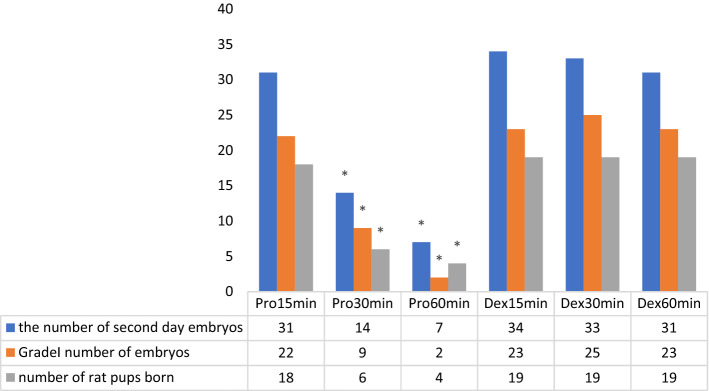
Comparison of the number of second day embryos between groups, the number of grade 1 embryos, and the number of female and male offspring born. *Pro15min* Propofol subgroup (0–15 min), *Pro30min* Propofol subgroup (15–30 min), *Pro60min* Propofol subgroup (30–60 min), *Dex15min* Dexmedetomidine (0–15 min), *Dex30min* Dexmedetomidine (15–30 min), *Dex60min* Dexmedetomidine (30–60 min). There was no fertilization in MI oocytes in groups Pro15min and Pro30min. The groups were compared using the Kruskal Wallis test; paired comparisons were made using the Mann Whitney U test for the different parameters (* P < 0.05).

**Figure 3:**
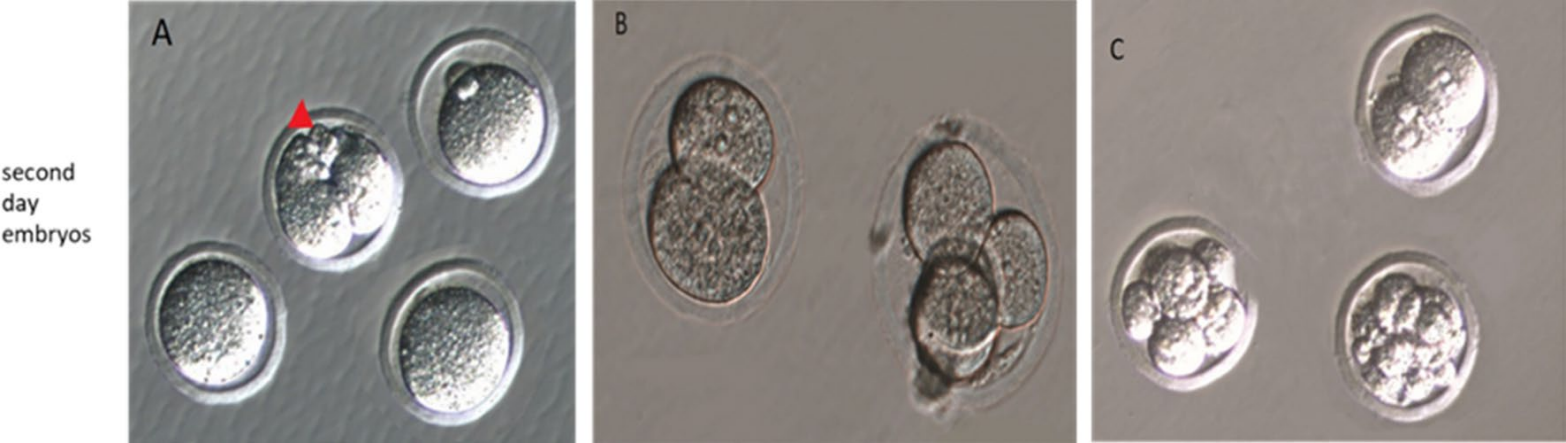
Images of the second day embryos belonging to the groups. (**A**) Propofol subgroup 3 (Pro60min); only one out of four oocytes is fertilized; fragmentation is observed in the fertilized embryo (Grade 2 quality). The red arrowhead shows fragmentation. (**B**) Propofol subgroup 1 (Pro15min) blastomeres of equal size and good quality embryos without fragmentation (Grade1 quality). (**C**) Dexmedetomidine subgroup 1 (Dex15min), high division rate and high-quality embryos without fragmentation are seen (Grade 1 embryo).

In this study, it was observed that the number of SDEs (Fig. 3), grade 1 embryos and the pups in the Pro15min subgroup were found to be statistically higher than the Pro30min and Pro60min subgroups (Fig. 2).

When SDEs, grade 1 embryos and pup numbers were compared between the Pro15min and Dex15min subgroups, there was no difference, but significant differences were observed between the Pro30min and Dex30min subgroups (P = 0.019, P = 0.015 and P = 0.017, respectively). A similar difference was found when the Pro60min and Dex60min subgroups were compared (P = 0.015, P = 0.017 and P = 0.011, respectively) (Fig. 3).

## Discussion

This study was conducted to evaluate the sensitivity of rat oocytes and embryos to anesthetics in order to observe the biological response for clinical studies on the effect of using propofol and dexmedetomidine as anesthetic agents on assisted reproductive technology results. In terms of providing effective conscious sedation and analgesia, Kwan et al. concluded that evidence from 21 randomized controlled trials did not support one particular method or technique over another during and after oocyte recovery^1^.

Due to the structure of sperm in humans, it is possible to obtain successful results with the ICSI procedure, especially if expert embryologists perform the procedure. However, the acrosome sperm structure is not compatible with the ICSI technique, as stated in rats and mice studies reported in published literature^10^. Therefore, the IVF technique was preferred over ICSI in this study. In addition, there are studies in the literature showing that there is no difference between fertilization rates, embryo quality and pregnancy rates in studies on IVF and ICSI^11,12^. In this current study, MII oocytes were primarily preferred for the IVF procedure. Studies have previously reported that incubated oocyte maturation completed in the MI phase produces a certain percentage of good quality embryos after fertilization^13,14^. In this current study, the incubation and maturation of oocytes were completed in the MI phase. The study included those that completed their maturation and turned into MII oocytes for IVF. Likewise, GV oocytes were incubated for maturation. However, we did not include the rare fertilized oocytes formed after this incubation. The successful results in our fertilization groups, especially in the Dex subgroups, were similar to the IVF-ET results in published literature.

With modern IVF technologies and with an expert operator, a human pick up lasts a few minutes^15^. However, the oocyte retrieval process takes longer in patients who develop too many follicles—such as polycystic ovarian patients—compared to other patients. As body mass index increases, the mean time spent in the operating room significantly increases^16^. To the best of our knowledge, this is the first study comparing the effect of dexmedetomidine and propofol on IVF results.

Previously published data have suggested a dose-dependent and time-dependent toxic effect that propofol has on the fertilization rates of oocytes^7,16–18^. These authors concluded that a propofol-based anesthetic technique resulted in significant concentrations of this agent in the follicular fluid that was related to the dose of propofol administered and the duration of its use^4,19^. Exposure of unfertilized oocytes to propofol results in a high degree of parthenogenetic activation^20^.

These observations point to a possible effect of anesthetic drugs on oocyte physiology and embryo development, and the quality and pregnancy outcomes after embryo transfer, which have not been sufficiently investigated so far. In this present study, the duration of anesthesia was negatively correlated with fertilization rates in the propofol group. It was observed that the negative effects of propofol on fertilization rate (FR), the number and quality of embryos, and the number of offspring obtained after embryo transfer, increased in direct proportion to the time spent under anesthesia (Table 1). The fact that this situation was not observed in terms of the number of oocytes obtained suggests that this negative effect of propofol is due to the exposure during the oocyte recruitment. We found a higher dependency between longer durations of anesthesia (> 30 min) and embryo quality, FR and the number of offspring obtained after embryo transfer (Figs. 1, 2). As the time spent under anesthesia with propofol increased, the negative effects on fertilization, embryo development and the number of offspring obtained after embryo transfer increased even more. In contrast to the present study, time-dependent toxic effects of propofol on fertilization rates have been reported in mice^7^. Tola^21^ concluded that the duration of anesthesia should be kept less than 30 min due to an association between the duration of the anesthesia and the lower implantation and clinical pregnancy rates. In this study, when the subgroups were compared on the basis of 0–15 min of administration for both agents, no difference was observed. However, the difference between the results of both agents was very prominent for the subgroups where 16–30 min and 31–60 min of exposure were evaluated. This result supports the idea that the shorter the exposure time, the less the expected side effects are. This suggests that propofol administration for anesthesia should be applied for as short a time as possible.

However, there are studies showing no such side effects for propofol^17^. Ben-Shlomo et al. concluded the time spent under anesthesia, as well as the total dose of propofol administered, are not associated with fertilization and embryo quality^18^.

Elnabtity et al. concluded that dexmedetomidine, used as a part of conscious sedation for oocyte retrieval, offered a favorable analgesic technique during and after the procedure with significantly less requirements than removing propofol intraoperatively^22^. It was observed that dexmedetomidine protects the ovarian tissue of the rat from ischemia and reperfusion (I/R) injury. Another study showed that dexmedetomidine has a protective effect on ovarian tissue against oxidative stress caused by pneumoperitoneum^23^. It is hypothesized that this protective effect of dexmedetomidine is mediated by the α-2 adrenergic receptors^24^. Gu et al. showed that dexmedetomidine was able to decrease lung injury caused indirectly by kidney I/R via α2-adrenoceptor-related and independent mechanisms^25^. This current study observed that the dexmedetomidine group was better than the propofol group in terms of the number and quality of embryos obtained. When dexmedetomidine subgroups were evaluated, there were none of the adverse effects observed in propofol subgroups with increased time spent under anesthesia and on FR, embryo number and quality, and the number of offspring obtained after embryo transfer. When evaluated in terms of the number of offspring obtained after embryo transfer, the dexmedetomidine group was superior to the propofol group (Fig. 2).

In this present study, no statistical differences were observed between embryo numbers and their quality, FR and the number of offspring obtained after embryo transfer of the dexmedetomidine subgroups (Fig. 2). This result showed that the dexmedetomidine exposure time does not affect the embryo number, quality and the number of offspring obtained after embryo transfer. This is thought to be the result of the protective effect of the dexmedetomidine and α-2 adrenergic receptors on ovarian tissue shown previously.

## Conclusions

As the exposure time to propofol used as an anesthetic agent for TVOR procedure increases, the number of embryos, their quality or the number of pups obtained after transfer decreases. This situation was not observed with the use of dexmedetomidine. The dexmedetomidine use had no adverse effect on the number of embryos, their quality or the number of pups obtained after transfer. When the results of this study were evaluated, it was thought that if propofol is used in TVOR applications, the procedure should be completed as soon as possible to reduce the exposure time to avoid the negative effects that may occur.

## Materials and methods

The study was conducted in Sakarya University’s SÜDETAM laboratory under the authority and approval of Sakarya University’s experimental animal ethics committee on 06/01/2021 under decision No: 06. All experiments were performed in accordance with ARRIVE guidelines, and all procedures were performed in accordance with the relevant guidelines. Twenty-four adult female Sprague Dawley rats (weight 200–250 g; age 65–75 days) were provided by the Sakarya University Animal Reproduction Center and housed in groups with ad libitum food and water in the Animal Laboratory of Sakarya University. The holding room was maintained at a room temperature of 22 ± 2 °C with humid conditions (45–55%) and a 12 h light/day cycle.

### Study design

The stage of the rats’ estrous cycle was determined by performing daily vaginal smears after acclimation. Rats determined to have at least three consecutive 4-day estrous cycles were prepared for in vitro fertilization (IVF). All the rats were subjected to the IVF protocol to create hyperstimulation.

### Stimulation and collection of oocytes

Twenty-four rats were prepared according to the stimulus protocol. Both ovaries were stimulated through the intraperitoneal (i.p.) route in the female rats. For the first injection, using an intraperitoneal injection of 150–300 internal units (IU)/kg of pregnant mare serum gonadotropin (CHRONOGEST/PMSG, Intervet, Istanbul, Turkey) was followed approximately 48 h later by 150–300 IU/kg of human chorionic gonadotropin (hCG; Gonatropin, CHORULON, Intervet, Istanbul, Turkey). At 17–19 h after hCG administration, 15 IU of Pregnant Mare Serum Gonadotropin (PMSG) was administered^26^.

The oocyte collection process was divided into four steps: Step 1, administration of the anesthetic agent used in the study; Step 2, waiting for the required time—according to the working group—followed by euthanasia (cervical dislocation) to extract the oviduct; Step 3, oocyte collection from the oviduct and Step 4, incubation of the oocytes until insemination.

The rats were then randomly divided into two different groups:

Group 1 rats were intravenously administered propofol (Propofol 1% FRESENIUS, FRESENIUS Kabi, Istanbul, Turkey) at 100 mg/kg. Group 1 rats were randomly divided into three different subgroups and the oviduct collection procedure completed within 15 min for Group Pro15min (n = 4), within 16–30 min for Group Pro30min (n = 4) and within 31–60 min for Group Pro60min (n = 4).

Group 2 rats were intraperitoneally administered a 25 µg/kg dexmedetomidine injection (PRECEDEX 200 µg/2 ml, Abbott, Istanbul, Turkey). Group 2 rats were also randomly divided into three different subgroups and the oviduct collection procedure completed within 0–15 min for Group Dex15min (n = 4), within 16–30 min for Group Dex30min (n = 4) and within 31–60 min for Group Dex60min (n = 4).

For oocyte pick-up, an aseptic technique was used to make a ventral midline incision to expose the reproductive organs and remove the oviducts. In this way the oocytes were collected from the removed ovaries. Oocytes were classified into the germinal vesicle (GV), the metaphase I (MI) and metaphase II (MII) stage. To compare meiotic progression during oocyte maturation in different systems, the average time for each stage of nuclear progression was calculated. This method was previously described by Sirard et al.^27^.

To incubate the oocytes, Human Tubal Fluid (HTF) medium (Cat. No. 90166, Irvine Scientific, USA) was cultured for one day before placing in an incubator at 37 °C in a 5% CO_2_ environment. Sperm collection and preincubation processes for mature male rats (outbred rats aged 10 weeks to 10 months) before the oocyte collection were performed using established procedures previously described by Hino^26^. The sperm were transferred to a droplet of HTF medium and incubated at 37 °C under 5% CO_2_ in humidified air for preincubation.

HTF medium (Cat. No. 90166, Irvine Scientific, USA) was used for sperm preincubation, fertilization and embryo transfer. Embryos were washed by passing through four such droplets which were kept at 37 °C under 5% CO_2_ in humidified air overnight.

The initial concentration of capacitated sperms was found to be approximately 1 × 10^6 ml^; the sperms were preincubated for approximately 15–60 min before being placed in fertilization drops and the final concentration of sperms was found to be approximately 4.5 to 6 × 10^5^ million/ml. Then sperms were transferred to the fertilization drops with oocytes.

Cumulus-oocyte complexes were transferred to culture media containing sperm and incubated in culture medium for 10 h at 37 °C and 5% CO_2_. After 10 h of incubation, the oocyte cumulus complexes were removed from the cumulus cells by taking the medium containing 0.1% Hyaluronidase. Oocytes with 2 pronuclei (2PN) and at least one sperm tail in the ooplasm were considered fertilized under an inverted microscope for fertilization control^28^.

Approximately 10 h after the IVF procedure, the oocytes were washed with HTF medium, transferred to the culture medium, and taken into an embryo culture. Fertilization control at 10 h after IVF was performed under an inverted microscope to identify any polyspermic fertilization or parthenogenetic embryos. Fertilization control was performed by controlling male pronucleus (MPN) formation of oocytes at 200 or 400 magnifications. The ooplasm was checked for an entire and enlarged sperm head and MPN. Because it is challenging to distinguish MPN from female pronucleus, oocytes with three pronuclei, two pronuclei and a second polar body or without a second polar body were considered MPN. Oocytes with a female pronucleus and a second polar body, or oocytes with two female pronuclei without a second polar body were considered activated^29^. At the 20th hour of embryo culture, 2-cell embryos were counted and embryos graded. These 2-cell embryos were also considered to be fertilized embryos^26^.

Then, fertilization was checked and the fertilized oocytes were washed and transferred to culture drops, and the resulting embryos were monitored up to transfer^30^. The total number of cells was counted as an embryo evaluation criterion: the ratio of blastomeres size and fragmentation rates, as well as the number of blastomeres, were used as described by Ahumada^31^.

### Cryopreservation and thawing of embryos

After the collection of oocytes, female rats were sacrificed without being used for embryo transfer. Female rats from the same source and at the same age as the sacrificed female rats were used for transfer^26^. Cryopreservation and thawing of second day embryos were performed using established procedures previously described by Nakao^32^. Embryos were stored frozen for 1–3 weeks. A survival rate of 94% was found for cryopreserved embryos after thawing.

### Transfer of embryos into oviducts

Embryo transfer was performed using previously described procedures by Nakagata^33^. Pseudo pregnant female rats were used that had been mated with vasoligated males. All pseudo pregnant female rats were anesthetized by an intramuscular administration of 50 mg/kg of ketamine hydrochloric acid (KETALAR; Eczacibasi Warner- Lambert Ilac Sanayi, Levent, Istanbul, Turkey) and 7 mg/kg of xylazine hydrochloric acid (ROMPUN, Bayer Sisli, Istanbul, Turkey). An aseptic technique was used to make a ventral midline incision to expose the reproductive organs and the oviducts. Then, the ovaries and oviducts were exposed and fat tissue near the ovaries was clamped for observation. Next, the embryos (sandwiched by air bubbles) were drawn into a capillary. The rats gave birth 20–21 days after surgery, and the numbers of pups were counted.

### Statistics

Statistical analyses were performed using SPSS 24.0 statistical software (SPSS Inc. and Lead Tech. Inc. Chicago. USA). The Kolmogorov–Smirnov test was used for normally distributed data. The Kruskal Wallis test was used for numerical data of the subgroups that did not show a normal distribution. Intergroup evaluations for statistically different parameters were performed using the Mann–Whitney *U* test and comparing them in pairs. Results are given as mean ± standard deviation. For all statistical analyses, a two-tailed p value < 0.05 was considered statistically significant.
